# Supplementary material for: NCBP3 positively impacts mRNA biogenesis
Source: Nucleic Acids Res. 2020 Sep 22;48(18):10413–27. doi: 10.1093/nar/gkaa744 (PMC7544205; doi:10.1093/nar/gkaa744)
Supplement: gkaa744_Supplemental_Files [file gkaa744_supplemental_files.zip › Supplemental Figures and Tables.pdf]

Supplemental figures and tables

Supplemental Figure S1. Related to Figure 1.

Supplemental Figure S2. Related to Figure 2.

Supplemental Figure S3. Related to Figure 3.

Supplemental Figure S4. Related to Figure 4.

Supplemental Figure S5. Related to Figure 5.

Supplemental Figure S6. Related to Figure 6.

Supplemental Table S1. Composition of extraction solutions used for the interaction screen. Related to Figure 1 and 2, and experimental procedures.

Supplemental Table S2. See Supplemental_Table_S2.xlsx. Stoichiometric abundance of proteins enriched with NCBP1, 2 and 3 in the IP screen. Related to Figure 1, Figure 2 and Supplemental Figure 2. Detailed description of the table is included in the spreadsheet.

Supplemental Table S3. Statistics of the RNA-seq library. Related to Figure 5.

Supplemental Table S4. BACs used for the cell lines. Related to experimental procedures.

Supplemental Table S5. List of siRNAs used. Related to experimental procedures.

Supplemental Table S6. Antibodies used for Western blots. Related to experimental procedures.

Supplemental Table S7. Antibodies used for immunofluoresence. Related to experimental procedures.

Supplemental Table S8. Primers used for qPCR. Related to experimental procedures.

Supplemental Figure S1

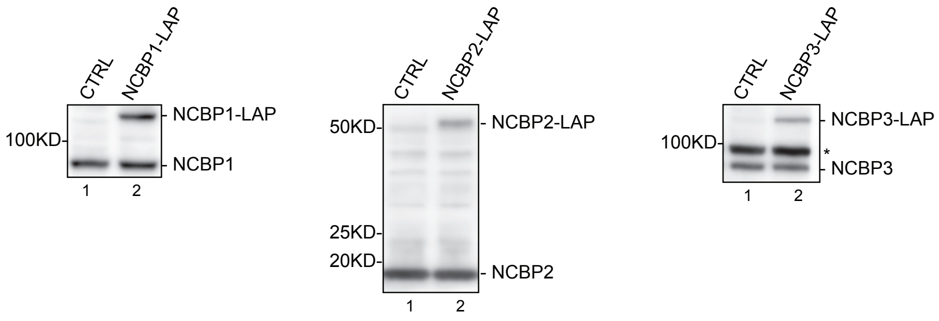

Supplemental Figure S1. Related to Figure 1.
Western blotting analysis of both endogenous and LAP-tagged NCBP proteins expressed in HeLa cells, using antibodies targeting NCBP1 (left), NCBP2 (mid) or NCBP3 (right). For each blot, lane 1 shows a non-tagged NCBP control. Asterisk indicates unspecific protein band.

Supplemental Figure S2

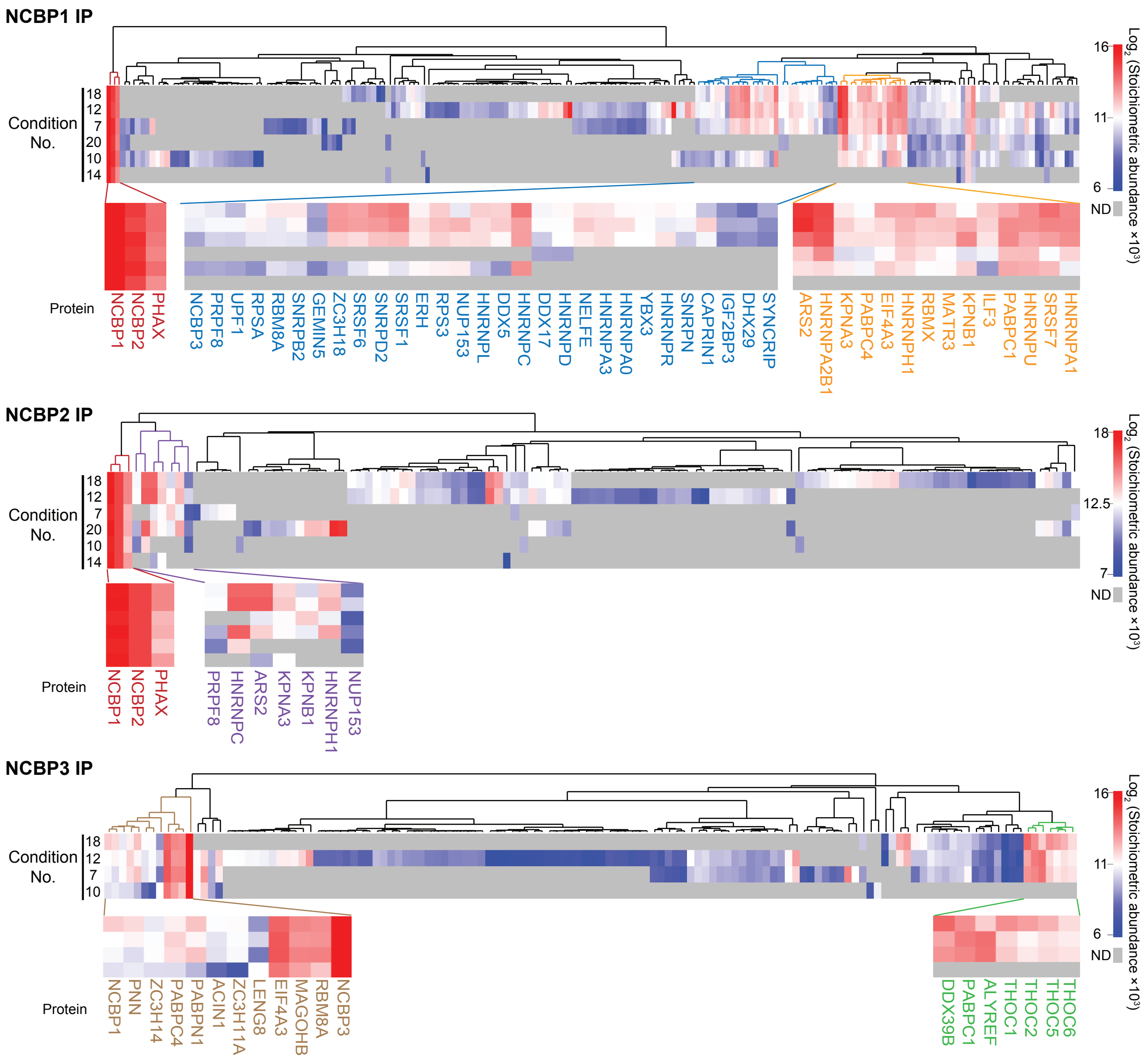

Supplemental Figure S2. Related to Figure 2.
Heatmap clustering of Euclidean distances showing log2 transformed stoichiometric abundances of NCBP1 (top), NCBP2 (mid) and NCBP3 (bottom) IPs. Missing values (ND) were imputed with 0 for the clustering. The heat colour range is shown on the right. Clusters where most proteins have abundance higher than the midpoint (in the red to white colour range) are highlighted, and zoomed-in versions with gene names are shown below the full heatmaps.

Supplemental Figure S3

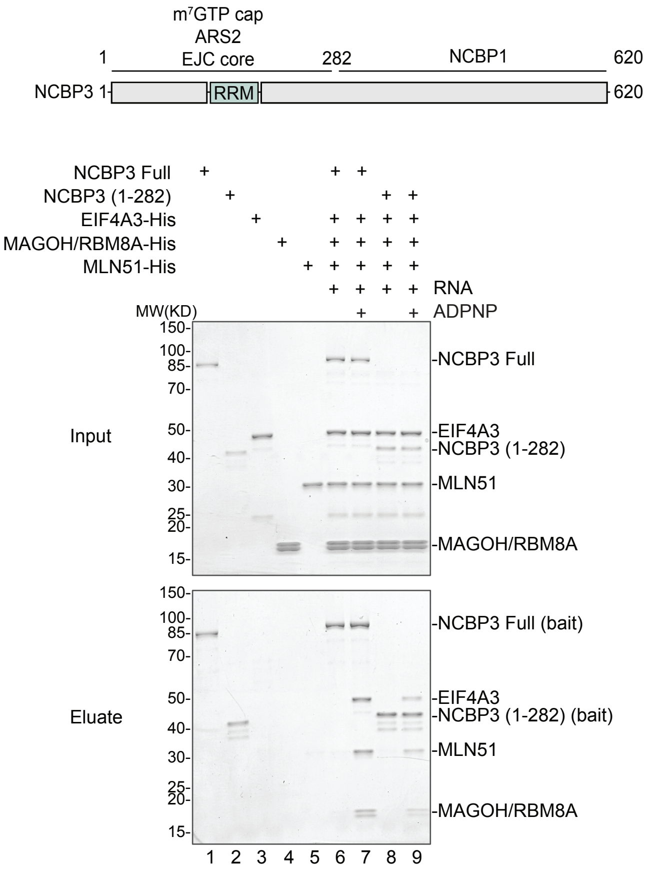

Supplemental Figure S3. Related to Figure 3.

Coomassie stained SDS-PAGE showing the results of protein binding assays of NCBP3 with EJC core components added as indicated on the top. Recombinant full length or N-terminal (1-128) NCBP3 (see schematics on the top) was incubated with EJC core components EIF4A3, MAGOH/RBM8A as a heterodimer and the selor domain of MLN51, and complemented with single-stranded biotinylated RNA with/without ADPNP. Pull-down assay were performed with the protein mix and m7GTP Sepharose.

Supplemental Figure S4

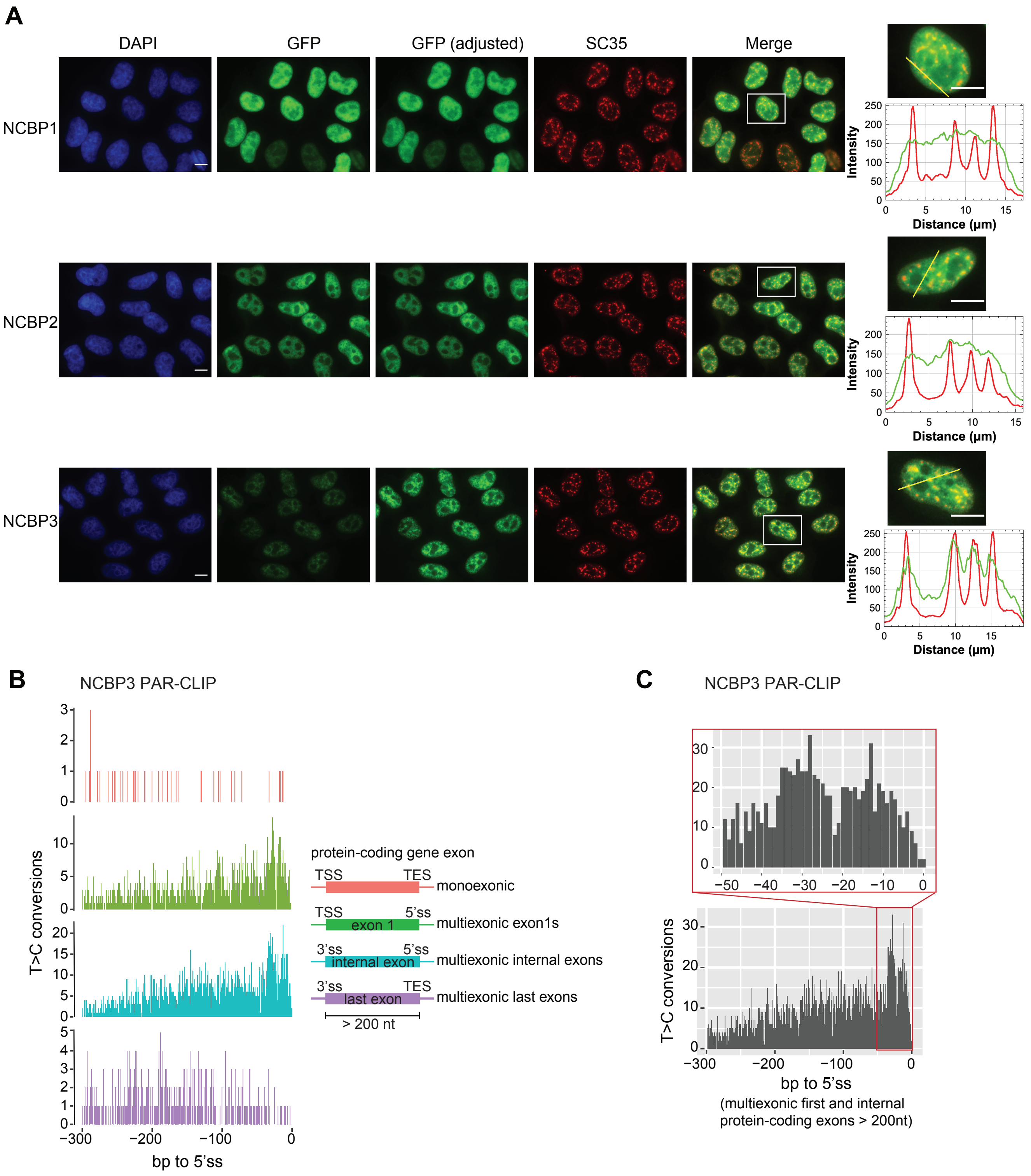

Supplemental Figure S4. Related to Figure 4.
(A) Immunolocalization microscopy of LAP-tagged NCBP1, NCBP2 and NCBP3 with SC35, using antibodies targeting GFP and SC35. DAPI, SC35 and NCBP3 signals are displayed as in Figure 4A. GFP brightness/contrast was set similarly across all NCBPs (second column) and also adjusted to achieve better visualization of individual NCBPs (third column). Merges were performed between adjusted GFP- and SC35-signals and signal scans were performed as in Figure 4A. Scale bars, 10 µm.
(B) NCBP3 PAR-CLIP T>C conversions on monoexonic exons as well as first, internal and last exons of multiexonic transcripts.
(C) A zoomed-in version of NCBP3 PAR-CLIP T>C conversions over multiexonic first and internal protein coding exons.

Supplemental Figure S5

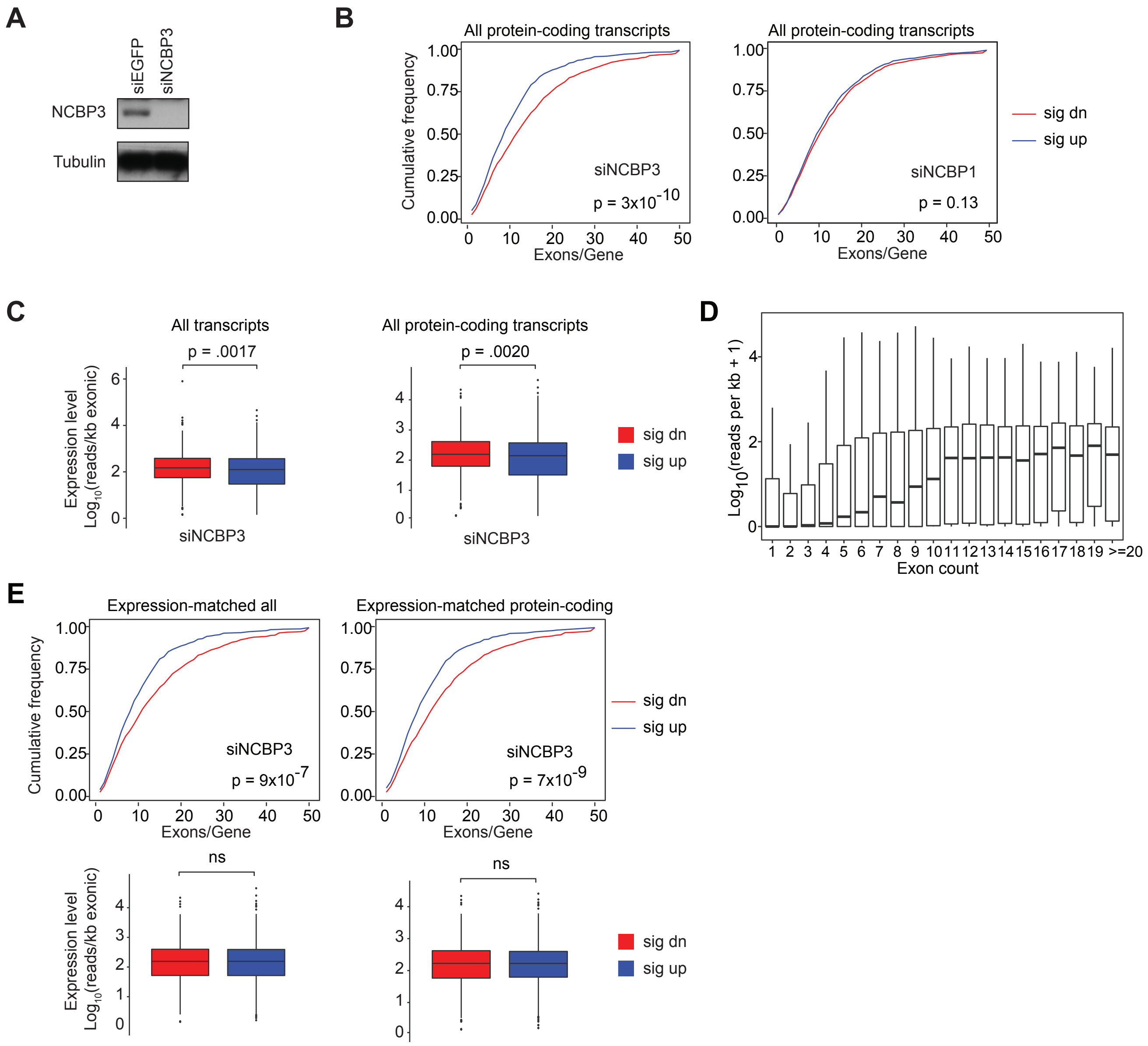

Supplemental Figure S5. Related to Figure 5.

(A) Western blotting analysis showing the depletion efficiency of NCBP3.

(B) Cumulative frequency plot of exon numbers in upregulated (blue) and downregulated (red) transcripts upon NCBP3 (left) and NCBP1 (right) depletion, as in Figure 5C and 5D, but for all differentially expressed protein coding transcripts.

(C) Expression level comparison between significantly up- and down-regulated transcripts. Expression levels per transcript were computed as mean read coverages per kb of exonic parts of transcripts for significantly up- (blue) and down-regulated (red) transcripts as in Figure 5C. The P value from a Mann-Whitney U-test comparing expression between significantly up vs. downregulated transcripts is indicated.

(D) Boxplot showing the gene expression levels (log10 transformed reads per kb) for transcripts grouped by their exon counts.

(E) Analysis of the correlation between exon numbers and changed expression upon NCBP3 depletion as in Figure 5C, but for an equal-sized subset of expression-matched transcripts (n=675, on the left panel) and expression-matched protein-coding transcripts (n=603, on the right panel), with the matched expression shown in the boxplots in the lower panel. The cumulative frequency plots and p values are obtained as described in Figure 5C.

Supplemental Figure S6

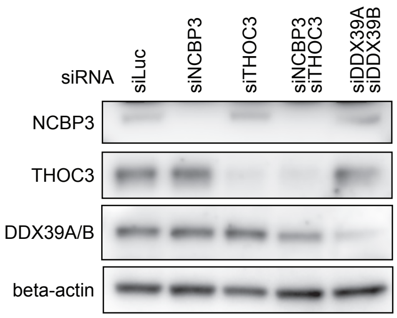

Supplemental Figure S6. Related to Figure 6.
Western blotting analysis showing protein depletion efficiencies with indicated siRNAs.

Supplemental Table S1 Composition of extraction solutions used for the interaction screen. Related to Figure 1 and 2, and experimental procedures.

Condition No.	Composition
7	20 mM HEPES-Na, pH 7.4, 150 mM NaCl, 5mM d-BCHAP
10	20 mM HEPES-Na, pH 7.4, 300 mM NaCl, 0.5mM Sarcosyl
12	20 mM HEPES-Na, pH 7.4, 150 mM NaCl, 0.5% TRIT X100
14	20 mM HEPES-Na, pH 7.4, 50 mM MgCl2, 0.5% TRIT X100
18	125 mM NH4 Acet, pH 7, 100 mM NaCl, 1% TRIT X100
20	20 mM TRIS-Cl, pH 8, 125 mM Na3 Cit, 1% TRIT X100

Supplemental Table S3 Statistics of the RNA-seq library. Related to Figure 5.

Library	Total reads	Trimming and read quality control		Mapping	
		read pairs remaining		uniquely aligned, proper read pairs	
siEGFP_1	41332086	38139659	(92.28%)	29984813	(78.62%)
siEGFP_2	52427151	48776569	(93.04%)	37667679	(77.22%)
siEGFP_3	40868991	37865523	(92.65%)	23137272	(61.10%)
siNCBP1_1	37239952	34934956	(93.81%)	27799828	(79.58%)
siNCBP1_2	41266225	38296661	(92.80%)	24757893	(64.65%)
siNCBP1_3	35493503	33126807	(93.33%)	25686591	(77.54%)
siNCBP3_1	45221352	42090840	(93.08%)	31615218	(75.11%)
siNCBP3_2	34333229	31658743	(92.21%)	21747355	(68.69%)
siNCBP3_3	45619227	42397189	(92.94%)	32942139	(77.70%)

Supplemental Table S4 BACs used for the cell lines. Related to experimental procedures.

Cell line	Gene Name	BAC ID	Tagging cassette	Ensembl ID
CTRL-LAP	-	CTD-3000G10	N-terminal	-
NCBP1-LAP	NCBP1	RP11-17A21	C-terminal	ENSG00000136937
NCBP2-LAP	NCBP2	HS.E139.A23	C-terminal	ENSG00000114503
NCBP3-LAP	NCBP3	RP11-118B14	C-terminal	ENSG00000074356
ALYREF-LAP	ALYREF	RP11-634L10	C-terminal	ENSG00000183684

Supplemental Table S5 List of siRNAs used. Related to experimental procedures.

Target	Sequence (sense 5'-3')
CTRL (Luciferase)	CUUACGCUGAGUACUUCGAdTdT
NCBP1	GGAAAGGAGUUGUACGAAAdTdT
NCBP2	GGCCAGAACUUGAGUAUUUdTdT
NCBP3	UCAGCGGGACGUGAUCAAGAAdTdT
DDX39A	AAAGGCCUAGCCAUCACUUUUdTdT
DDX39B	AAGGGCUUGGCUAUCACAUUUdTdT
ALYREF	UGGGAAACUGCUGGUGUCCAAdTdT
EIF4A3	AGACAUGACUAAAGUG GAAdTdT
RBM8A/Y14	CGCUCUGUUGAAGGCUGGAdTdT
THOC3	GUGUGAGUCUCCGACCUUCdTdT

Supplemental Table S6 Antibodies used for Western blots. Related to experimental procedures.

Target	Origin	Source	Cat #/reference	Working dilution
NCBP1	Rabbit	E.Izzauralde		1:1000
NCBP2	Rabbit	E.Izzauralde		1:1000
NCBP3	Rabbit	Sigma	HPA013195	1:1000
GFP (B-2)	Mouse	Santa Cruz	sc-9996	1:1000
ALYREF	Rabbit	Abcam	Ab202894	1:10000
EIF4A3	Rabbit	H.Le Hir		1:1000
RBM8A/Y14	Rabbit	H.Le Hir		1:500
MLN51	Rabbit	C.L.Tomasetto	Degot et al., 2002	1:1000
MAGOH	Mouse	Santa Cruz	sc-56724	1:500
DDX39A/DDX39B	Mouse	Santa Cruz	sc-271395	1:500
HA	Rabbit	Abcam	ab9110	1:5000
THOC3	Rabbit	Sigma	HPA044009	1:500

Supplemental Table S7 Antibodies used for immunofluoresence. Related to experimental procedures.

Target	Origin	Source	Cat #/reference	Working dilution
NCBP3	Rabbit	Atlas Antibodies	HPA013195	1:500
SC35	Mouse	Abcam	ab11826	1:1000
GFP	Rabbit	Invitrogen	A-6455	1:1000
Mouse (Alexa Fluor 488)	Goat	Invitrogen	A11034	1:1000
Rabbit (Alexa Fluor 647)	Goat	Invitrogen	A21244	1:1000
Mouse, (Alexa Fluor 633)	Goat	Invitrogen	A21052	1:1000
Rabbit (Alexa Fluor 488)	Donkey	Invitrogen	A21206	1:1000

Supplemental Table S8 Primers used for qPCR. Related to experimental procedures.

Primer name	Sequence (5'-3')
PSMD2 Exon16_18.fw	GCACGTAATTCACACAACTGAC
PSMD2 Exon17.fw	CAGCTCCTGCCACTCCTTAG
PSMD2 Exon18.rev	GCTGCTGACATCATCTCCGT
ALT2 Exon1_3.fw	CGACCTCCCTAGGATTCTCA
ALT2 Exon2.fw	GTAGTGGTATCTGTGGCAGGAG
ALT2 Exon3.rev	CTCGCCATGTAAAGCCTGTCA
MRPL3 Exon3_4.fw	CATTACTTCAGGTACAAGAC
MRPL3 Exon3_5.fw	CATTACTTCAGAAAGCTACATCC
MRPL3_Exon5.rev	GCCTGGTTTAATTGCAGCAT
